# Predicting lung adenocarcinoma disease progression using methylation-correlated blocks and ensemble machine learning classifiers

**DOI:** 10.7717/peerj.10884

**Published:** 2021-02-16

**Authors:** Xin Yu, Qian Yang, Dong Wang, Zhaoyang Li, Nianhang Chen, De-Xin Kong

**Affiliations:** 1 State Key Laboratory of Agricultural Microbiology, Huazhong Agricultural University, Wuhan, Hubei, China; 2 Agricultural Bioinformatics Key Laboratory of Hubei Province, College of Informatics, Huazhong Agricultural University, Wuhan, Hubei, China

**Keywords:** Methylation correlated blocks, Ensemble model, Lung adenocarcinoma

## Abstract

Applying the knowledge that methyltransferases and demethylases can modify adjacent cytosine-phosphorothioate-guanine (CpG) sites in the same DNA strand, we found that combining multiple CpGs into a single block may improve cancer diagnosis. However, survival prediction remains a challenge. In this study, we developed a pipeline named “stacked ensemble of machine learning models for methylation-correlated blocks” (EnMCB) that combined Cox regression, support vector regression (SVR), and elastic-net models to construct signatures based on DNA methylation-correlated blocks for lung adenocarcinoma (LUAD) survival prediction. We used methylation profiles from the Cancer Genome Atlas (TCGA) as the training set, and profiles from the Gene Expression Omnibus (GEO) as validation and testing sets. First, we partitioned the genome into blocks of tightly co-methylated CpG sites, which we termed methylation-correlated blocks (MCBs). After partitioning and feature selection, we observed different diagnostic capacities for predicting patient survival across the models. We combined the multiple models into a single stacking ensemble model. The stacking ensemble model based on the top-ranked block had the area under the receiver operating characteristic curve of 0.622 in the TCGA training set, 0.773 in the validation set, and 0.698 in the testing set. When stratified by clinicopathological risk factors, the risk score predicted by the top-ranked MCB was an independent prognostic factor. Our results showed that our pipeline was a reliable tool that may facilitate MCB selection and survival prediction.

## Introduction

Lung adenocarcinoma (LUAD) is one of the leading causes of cancer-related death (Siegel, Miller & Jemal, 2018). The poor prognosis for LUAD patients is due to several factors, including late disease diagnosis and the lack of effective drugs. Even stage I LUAD patients who undergo potentially curative surgical resection are at high risk of death caused by recurrent disease, and there is a 5-year relapse rate of 35% to 50% (Hanagiri et al., 1999; Rivera et al., 2011). One explanation for low overall survival during the early stages could be the high risk of local recurrence and distant metastasis after treatment. Moreover, in the absence of useful biomarkers, all stage I LUAD are pooled, making it more difficult to draw meaningful clinical conclusions (Sandoval et al., 2013). Therefore, developing and validating diagnostic biomarkers that can predict which patients are at the highest risk of relapse may help identify subgroups that can benefit from intensified systemic therapy with improved outcomes.

Obtaining reliable and quantitative measurements using the minimum number of markers is challenging, and more sensitive assays need to be developed. Despite recent progress, the molecular mechanisms underlying prognosis have not been explained in detail. In the search for new potential human cancer biomarkers, the hypermethylation of cytosine-phosphorothioate-guanine site (CpG) island sequences located in the promoter regions of tumor suppressor genes is gaining prominence (Guo et al., 2017; Hao et al., 2017). Several previous studies have analyzed the involvement of individual CpG sites (Zeng et al., 2017), methylation differences in tumors and cell lines (Capper et al., 2018; Sahm et al., 2017; Witt et al., 2018), and methylation differences between primary tumors and normal lung tissue (Diaz-Lagares et al., 2016). These studies have helped to decipher biological differences across these systems but did not define prognostic parameters. Recent research has shown that adjacent CpG sites in the same DNA strand may be modified by methyltransferases and demethylases (Burger et al., 2013; Guo et al., 2017). We referred to these adjacent CpG methylation stretches as methylation-correlated blocks (MCBs) or methylation haplotype loads, and they are similar to haplotype blocks of adjacent single nucleotide polymorphisms in DNA sequence variations (Ardlie, Kruglyak & Seielstad, 2002). Additionally, methylated blocks can be characterized using summary statistics in sliding windows that contain several CpGs (Burger et al., 2013; Feldmann et al., 2013; Tong et al., 2018).

These MCBs have the potential to substantially improve the accuracy of diagnosis prediction (Guo et al., 2017; Hao et al., 2017). Previous research applied algorithms such as the LASSO and the Cox proportional hazards model to distinguish between adjacent normal and tumor samples. However, the remarkably high clustering performance when using large-scale methylation data in blood plasma is controversial (Seoighe, Tosh & Greally, 2018) and this method should only be used an alternative option for clinical diagnosis. A suitable model using a methylation haplotype load that can provide acceptable survival predictions has not been discovered, which hinders possible clinical applications.

There is a demand for an advanced model that can identify patterns underlying CpG changes in such MCBs and disease progression, as methylation changes are correlated with disease-free survival (DFS) (Capper et al., 2018; Liao et al., 2018). However, previous studies made predictions mainly based on the sum of methylation values of all intro CpGs in a MCB (Guo et al., 2017; Hao et al., 2017). Because the methylation peaks for intro CpGs in MCBs may also be changed, there is a limited number of single, mean-based predictive MCB models. These changes were reflected in two MCB values, namely the compound methylation value of the whole MCB and the individual methylation value of the intro CpGs. Many different machine learning algorithms and techniques have been developed to detect these changes. The models have different levels of effectiveness and specialties for selecting representative CpGs across a bulk of correlated parameters in the survival models. For example, the elevated performance of support vector regression (SVR) is used to determine disease progress using the methylation values of all the intro CpGs (Das, 2010), and the elastic-net model is used to select the most optimal panel or features of the intro CpGs for survival. Moreover, a combination of these algorithms could also be an option (Choubin et al., 2019; Guo & Sui, 2019; Van Belle et al., 2011). By using a form of regression as the secondary classifier, most stacking applications to date have improved performance over both the original classifiers and regression-mediated models (Sloutsky & Naegle, 2019). An ensemble model may more comprehensively reflect the variety of intro CpG changes in an MCB. Despite the promising results provided by these methods, predicting survival rates based on methylated regions that were extracted from tens of thousands of methylation profiles is a difficult task to execute.

**Figure 1:**
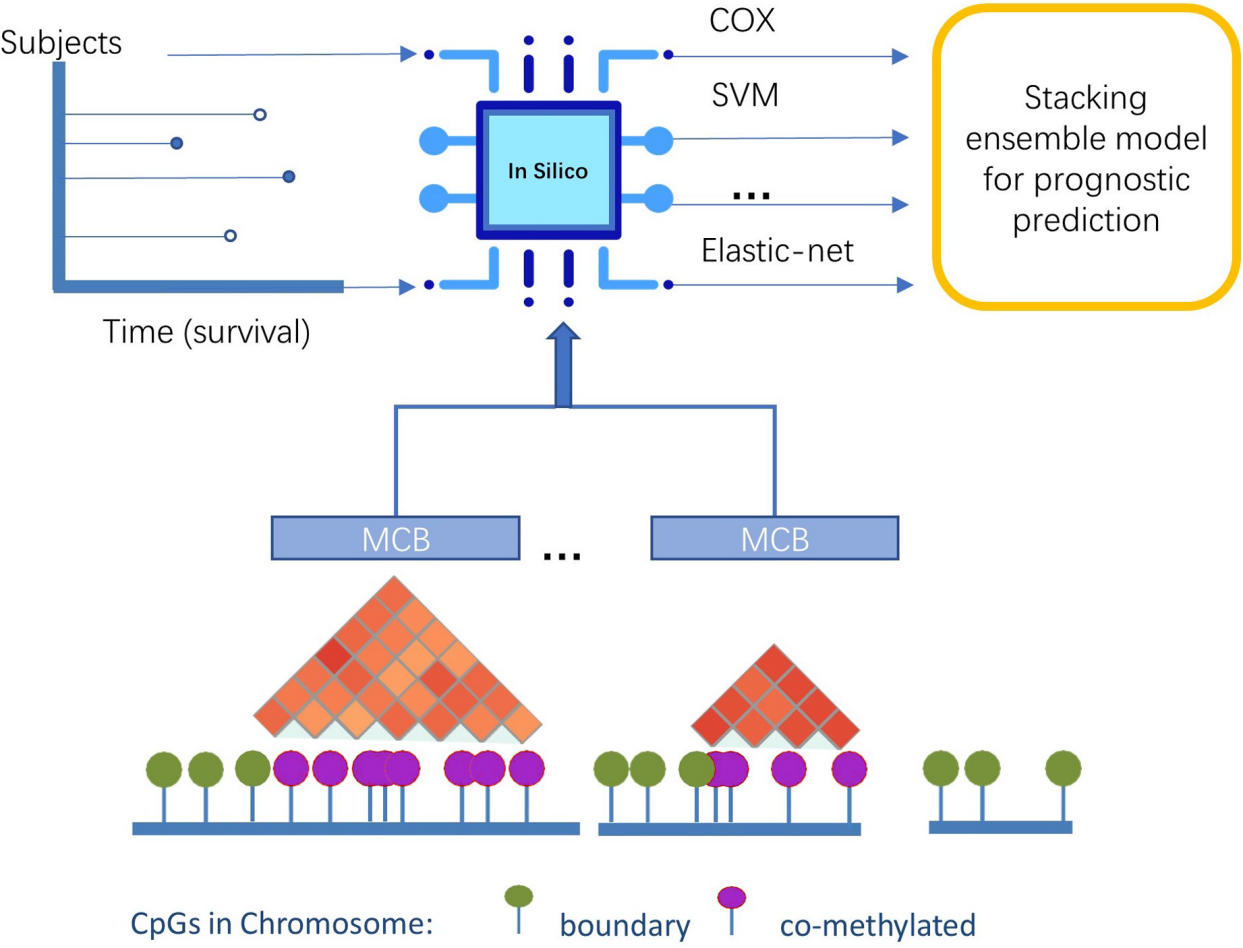
The overview of pipeline design.

In this study, we presented a novel pipeline named stacked ensemble of machine learning models for methylation-correlated blocks (EnMCB) that automatically finds methylation-correlated blocks and introduces them as signatures to build machine learning models (Fig. 1). We built the MCB-based classifiers using a panel of biologically and statically relevant CpGs. The ensemble model was also used because of its potential to enhance the performance of the prognostic models (Hao et al., 2017). These methods could easily be implemented using R and Bioconductor (Huber et al., 2015; Yu, 2020). Our results may also reveal deeper insights into the molecular markers of disease progression.

## Materials and Methods

### Data collection

We analyzed and developed a methylation signature using Illumina Infinium Human Methylation 450K BeadChip data obtained from the Cancer Genome Atlas (TCGA) and Gene Expression Omnibus (GEO). We extracted clinical information on the TCGA data from the Pan-Cancer Clinical Data Resource (Liu et al., 2018).

We downloaded the TCGA level-3 methylation and gene expression profile for LUAD from the RTCGA package (version 1.8.0, http://gdac.broadinstitute.org/). The GSE39279 methylation profiles (Sandoval et al., 2013) for LUAD in the GEO database were downloaded using the GEOquery package (version 2.46.15). The TCGA database included only one primary solid tumor sample per patient analysis.

To determine the DFS of LUAD profiles in TCGA, we defined a disease event as when a patient has a new tumor event, including local recurrence, distant metastasis, or new primary tumors at all sites. DFS was defined as the time before a disease event, measured from the date of surgery to the date of the disease event. Patients were designated as censored cases when their days to last contact met the criteria. Since new primary tumor events are not released in GSE39279, we defined a disease event for GSE39279 as when a patient has a local recurrence or distant metastasis. We used a dataset from TCGA as the training set. We randomly divided the GEO dataset into a validation set (1/3) and a testing set (2/3). The clinical characteristics of the samples in the two public datasets are summarized in Table S1.

### Data preprocessing

Methylation profiles were preprocessed using a chip description file. This chip description file, which includes CpG-based annotation files from the Illumina Infinium Human Methylation 450 K BeadChip, allows for direct mapping of CpGs to the corresponding genes (Ensembl IDs or gene symbols). These annotation files allowed us to extend gene annotation and labeling by providing a table that contains the gene symbols and other CpG characteristics in an R expression set.

Briefly, the degree of DNA methylation at each CpG was denoted as a *β* value, which we calculated as:

(1) \begin{eqnarray*}\begin{array}{@{}c@{}} \displaystyle {\mathrm{Beta}}_{i}= \frac{\mathrm{max} \left( {y}_{i, \mathrm{methy}}, 0 \right) }{\mathrm{max} \left( {y}_{i, \mathrm{methy}}, 0 \right) +\mathrm{max} \left( {y}_{i,\mathrm{unmethy}}, 0 \right) +\varepsilon } \end{array}\end{eqnarray*}

where *y*_*i*,methy_ and *y*_*i*,unmethy_ are normalized values of the methylated and unmethylated allele intensities, and ε is the constant offset (=100 generally) that was recommended by Illumina. There were a total of 485,577 CpG features. The beta value ranged from 0 to 1, reflecting the fraction of methylated alleles at each CpG in each sample.

We removed the CpG sites missing values or that were associated with known single-nucleotide polymorphism (SNP) loci, and replaced the CpG sites with a few missing values (<30%) with the mean value of the whole feature. A total of 391,513 features remained after preprocessing.

The *β* values were processed using noob background correction (Triche Jr et al., 2013), dye bias correction, and quality control (Zhou, Laird & Shen, 2017) procedures that followed TCGA’s data processing pipeline for Level 3 TCGA data (Weinstein et al., 2013). The GEO dataset was preprocessed using Genome Studio V2011.2 following previously described Illumina standard procedures (Sandoval et al., 2013). We further adjusted the *β* values to prepare the GEO datasets for batch effect using the Combat method (Zhang et al., 2018) referenced by TCGA. A sex discordance check was carried out using a chi-squared test.

### Identifying methylation-correlated blocks

We evaluated a genome-wide DNA methylation profile of 455 samples. To avoid any potential heterogeneity, we excluded 32 adjacent normal lung samples, two samples with no primary tumors, and three replicated samples from the original cohort of 492 samples in the LUAD TCGA database. All samples in the training set were used to partition the genome into blocks of tightly co-methylated CpG sites (MCBs) according to (Hao et al., 2017). We calculated Pearson correlation coefficients *r*^2^ between the *β* values of any two CpGs positioned within one kilobase, using an *r*^2^ cutoff of 0.8. We used a Pearson’s value of *r*^2^ < 0.8 to identify transition spots (boundaries) between any two adjacent markers that indicated uncorrelated methylation. Markers not separated by a boundary were combined into the MCB.

This procedure identified a total of 31,726 MCBs, with two to 45 CpG positions in each block. We also calculated the Pearson correlation coefficients between the two adjacent CpGs and the standard deviations.

### Linking methylation-correlated blocks markers to gene expression

The relative gene expressions were calculated using the RNAseq transcriptome data from TCGA database. Because of the wide variation of raw count values, we log2 transformed the values. We then identified genes with methylation values that correlated with varied associated gene expression levels using a Pearson correlation test. If the MCB covered multiple gene transcription start sites, we selected the gene that had the correlation coefficient with the greatest absolute value as the corresponding gene for this MCB. If no correlated gene was found, we set the result as “not available” (NA).

### Prognosis models for methylation correlation blocks

LUAD tumor samples were selected from patients with available DFS clinical data (not NA). We selected 455 methylation profiles from a total of 492 samples in TCGA, and a total of 155 out of 444 samples using the same criterion from the GEO dataset. We next assessed the prognostic utility of the methylation signatures for survival based on the selected data and using the ensemble model with two stages, which are described in 2.6.1 and 2.6.2, respectively.

#### Stage 1: prognosis models for CpGs and MCBs

For individual CpGs, we used univariate Cox proportional hazards regression to evaluate the independent prognostic factors (CpGs) associated with DFS. To enable quantitative analysis of the methylation patterns within individual MCBs across many samples, we needed a single metric to define the methylated pattern of multiple CpG sites within each block.

We calculated the arithmetic mean of all CpGs in each MCB, and used those arithmetic mean values for feature selection. We selected MCBs with at least five intro CpGs. Then, using the MCB arithmetic mean values, we carried out the embedded feature selection step (Laimighofer et al., 2016) with L1 regularization (threshold of *λ* = 0.01) based on Cox regression with partial likelihood deviances to assess the correlation of survival and to filter out unrelated MCBs. We collected the results for filtered MCBs to show their prediction capacity.

Apart from the arithmetic mean, we constructed three separate learning models as follows:

First, we constructed the classic Cox regression model *G*_*COX*_. The minimizing coefficients were defined using the ordinary least squares method. Second, we built the support vector regression *G*_*SVR*_ model using a linear kernel function (details can be found in Fouodo et al., 2018; Van Belle et al., 2011). Third, the elastic-net generalized Cox model *G*_*EN*_ (Friedman, Hastie & Tibshirani, 2010; Simon et al., 2011) was constructed following the Cox regression model. The negative log of the partial likelihood was penalized using an elastic-net penalty. This penalty eliminated the covariates of the intro CpGs (CpGs in MCBs) with low effect (threshold of minimum *λ*) on the predictive model.

The prediction values for all three models in the training set were determined using the 10-fold cross validation method. For predictions in the testing set, we used the median score in the training to generate the cutoff signature score. The parameters used for tuning the models, package information, and environment details are recorded in Table S5.

#### Stage 2: stacking ensemble model construction

All CpG risk scores were calculated in individual MCBs using Cox, SVR, and elastic-net models. We used the predictions as the compound methylation MCB values. We then constructed multi-model-based stacking ensemble classifiers (Simopoulos, Weretilnyk & Golding, 2018) to predict the survival of LUAD patients using feature-weighted linear stacking (Sill et al., 2009) as follows:

Seeking a blended prediction function $d \left( x \right) $ for multi-model-based classifiers *g*_*i*_ based on all the samples *x* ∈ 𝔛 with the formula

(2) \begin{eqnarray*}\begin{array}{@{}c@{}} \displaystyle d \left( x \right) =\sum _{i}{w}_{i}{g}_{i} \left( x \right) \end{array}\end{eqnarray*}

where *w*_*i*_ is a learned model weight in ℝ. In feature-weighted linear stacking, *f*_1_, *f*_2_, …, *f*_*j*_ represent a collection of *j* meta-feature functions to be used for stacking. The weight values *w*_*i*_ can be modeled as linear functions of the meta-features

(3) \begin{eqnarray*}\begin{array}{@{}c@{}} \displaystyle {w}_{i} \left( x \right) =\sum _{j}{v}_{ij}{f}_{j} \left( x \right) \end{array}\end{eqnarray*}

where *v*_*ij*_ is the learned weights. Equation (3) can be rewritten as

(4) \begin{eqnarray*}\begin{array}{@{}c@{}} \displaystyle d \left( x \right) =\sum _{i,j}{v}_{ij}{f}_{j} \left( x \right) {g}_{i} \left( x \right) . \end{array}\end{eqnarray*}

We vat optimized the problem by minimizing the loss function in Eq. (4). The learned weights *v*_*ij*_ can be estimated using the training set $x\in \tilde {\mathfrak{X}}$ with the formula

(5) \begin{eqnarray*}\begin{array}{@{}c@{}} \displaystyle {\mathrm{min}}_{v}\sum _{x\in \tilde {\mathfrak{X}}}\sum _{i,j}{ \left( {v}_{ij}{f}_{j} \left( x \right) {g}_{i} \left( x \right) -y \left( x \right) \right) }^{2}. \end{array}\end{eqnarray*}

Finally, we calculated the risk score for each patient based on the individual MCB models. The prediction values for the ensemble model in the training set were also determined using the 10-fold cross validation method.

### Survival analysis of the best stacking ensemble model

We then tested the univariate associations of the clinical pathological characteristics (Table S1) and each individual MCB model with DFS in the training and testing sets. We used rank product (RP) statistics (Koziol, 2010) to evaluate the performance of the stacking ensemble model:

(6) \begin{eqnarray*}\begin{array}{@{}c@{}} \displaystyle R{P}_{i}=\sum _{j=1}^{k}\log \nolimits \left( {R}_{ij} \right) \end{array}\end{eqnarray*}

where *i* = 1, …, *n*, represented each MCB, and *j* represented each model, i.e., the results of the area under the receiver operating characteristic (ROC) curve (AUC) that tested the associations between RFS and Cox, SVR, Elastic-Net and ensemble model predictions in the training and validation sets. Next, we ranked the summary scores of the prediction AUCs for each model, which formed *R*_*ij*_ = *rank*(AUC). Small rank values were marked for better results.

We used Cox proportional hazards analysis to evaluate the performance of the associations between the models and the DFS clinic factor. We further evaluated the performance of the models using ROC curves, followed by calculating the AUC (Kamarudin, Cox & Kolamunnage-Dona, 2017) and C-index values. For ranking methods, we mainly relied on AUC values, which may be more open to interpretation (Blanche, Kattan & Gerds, 2019), Kaplan–Meier survival analysis, and log-rank test.

We defined the cut-off value for the survival curve as the median value of the risk scores in the training set. We used bootstrap percentile method (Robin et al., 2011) to compare the AUCs for different models in the same MCB. We rounded AUC values to three decimal places. We used paired *t*-test to compare classifiers over multiple datasets (MCBs) (Demšar, 2006). We calculated the adjusted *p* values using the B.H. method (Benjamini & Hochberg, 1995). A *p* value <0.05 was defined as statistically significant. Figures were plotted using ggplot2 package in R and GraphPad Prism. Analysis for machine learning, AUC calculation, and identifying the methylation-correlated blocks were performed using the EnMCB package (http://www.bioconductor.org/packages/release/bioc/html/EnMCB.html) in Bioconductor (Huber et al., 2015; Yu, 2020). The source package and analysis code are freely available at GitHub (https://github.com/whirlsyu/EnMCB_analysis_for_lung_adenocarcinoma/).

## Results

### Identifying methylation correlation blocks

We used the DNA methylation microarrays within all primary LUAD (*n* = 455) to identify the methylation correlation blocks. Because of the underlying assumption that DNA sites in close proximity are co-methylated, we studied the degree of co-methylation across different DNA strands by using the well-established concept of genetic linkage disequilibrium. We applied a Pearson correlation method (using an *r*^2^ cutoff of 0.8) to quantify the co-methylation of close CpG sites. Then, we partitioned the genome into blocks of tightly co-methylated CpG sites.

**Figure 2:**
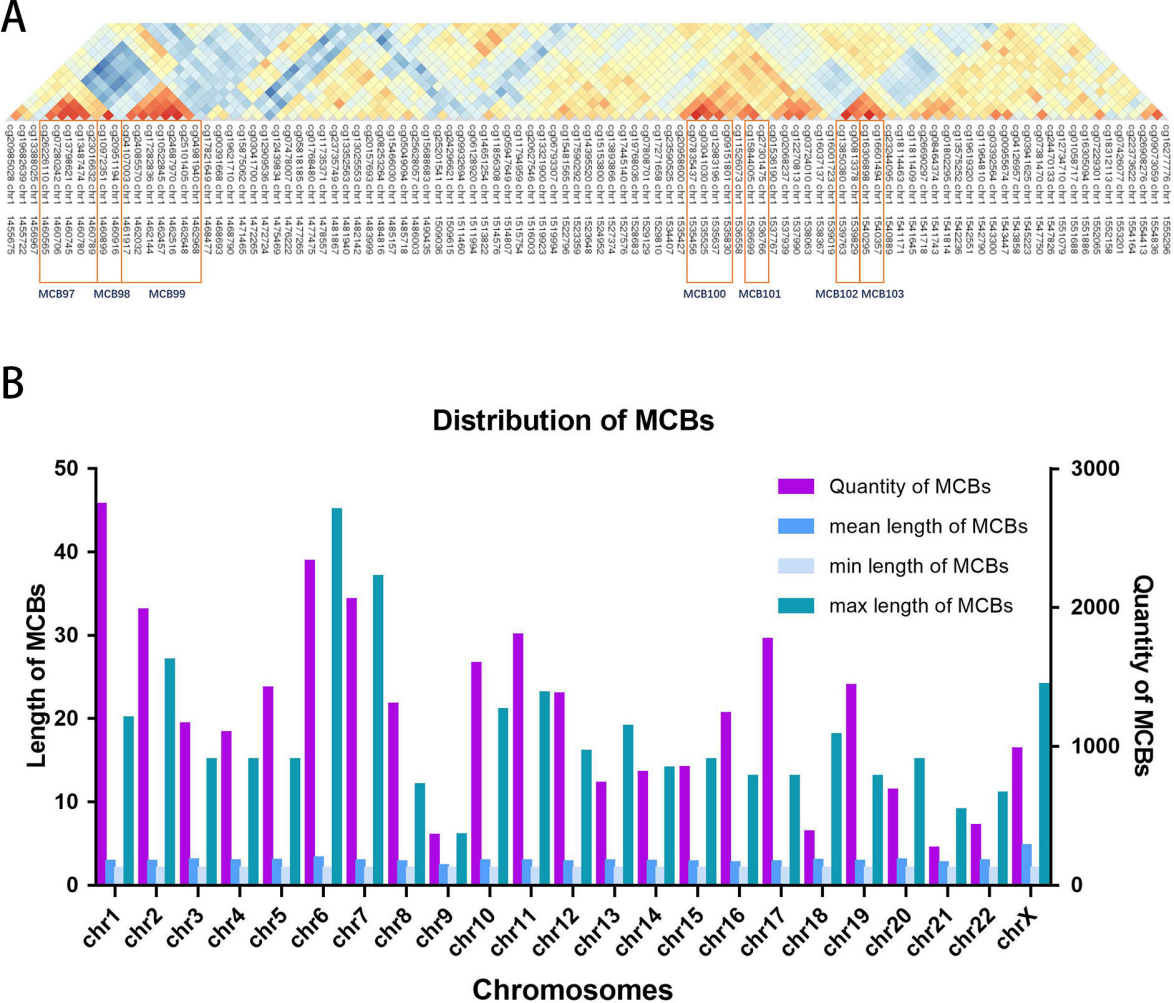
Distribution of MCBs in chromosome. (A) An example of seven MCBs found on chromosome 1. Red indicates strong correlation, while blue indicates weak correlation. Red rectangles indicate individual MCBs. (B) The quantity of MCBs in DNA from multiple (chromosomes 1-22, X) chromosomes.

Figure 2A shows the seven MCBs found on chromosome 1. Overall, we surveyed and found 31,726 MCBs that covered approximately 19 percent of the total CpG sites (there were 93,196 CpG sites in MCBs and 485,577 CpG sites).

The MCB quantities for the chromosomes are shown in Fig. 2B. The MCB quantities did not correlate with chromosome length, although chromosome 1 had the greatest quantity of MCBs. MCB lengths ranged from 3 bp to 4,283 bp and had an average of 193 bp (Fig. S1). The minimum number of CpG sites within the individual MCB was 2, while the maximum number was 45 (Fig. S2).

**Figure 3:**
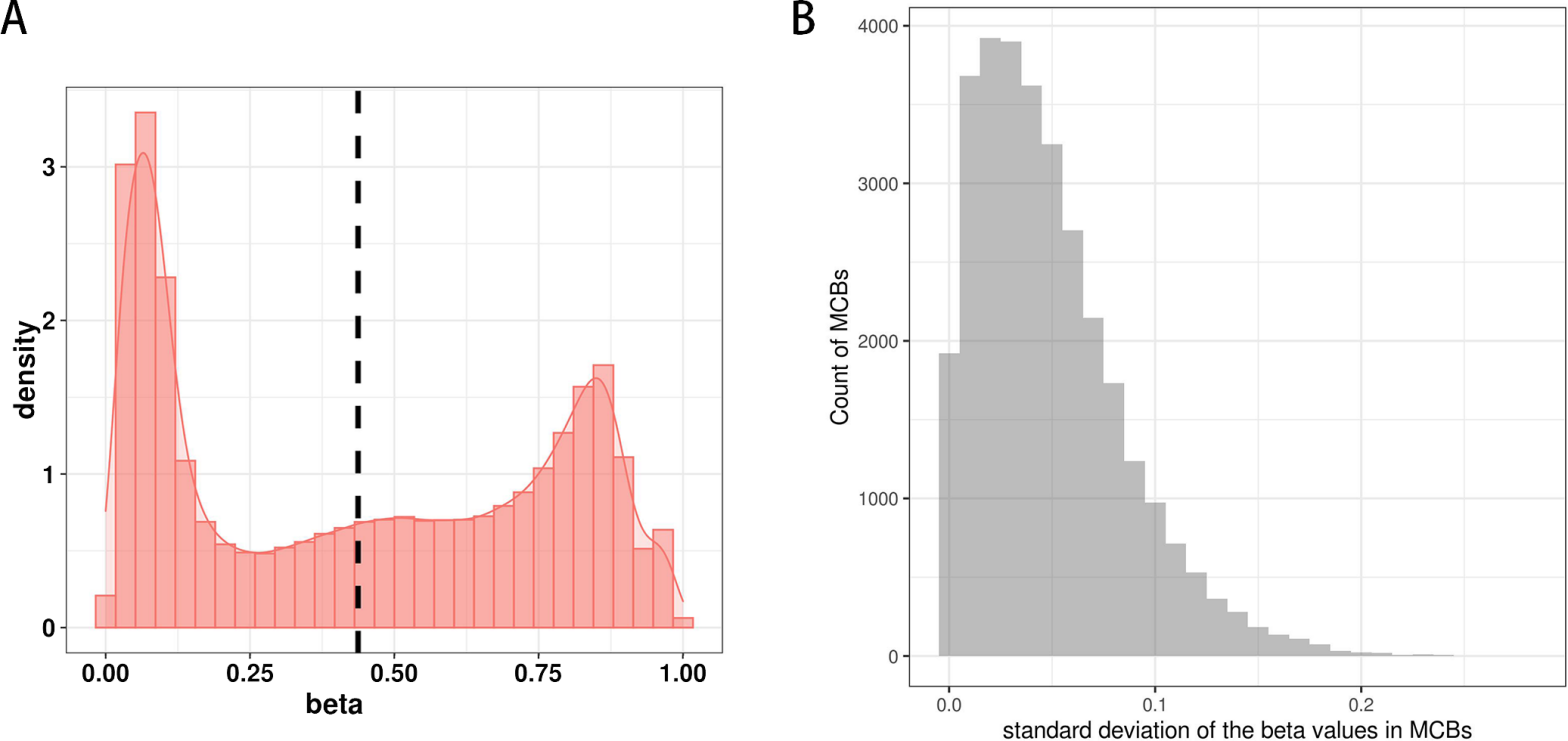
The distribution of CpG sites. (A) The distribution of *β* values of the CpG sites from one sample in the training set. (B) The distribution of standard deviation of mean values of the CpG sites in MCBs.

Figure 3A shows the distribution of *β* values for all CpG sites from one sample in the training set. The distribution of *β* values was consistent with the three reference standards (peaks), which showed low *β* values, intermediate *β* values, and high *β* values.

We next determined the methylation values within the MCBs. For most of them, we found similar *β* values across multiple CpG sites within one MCB, but we also observed a relatively high standard deviation for the mean *β* values of multiple CpG sites in individual MCBs. In some MCBs, the standard deviation was as high as 0.28 (Fig. 3C).

**Figure 4:**
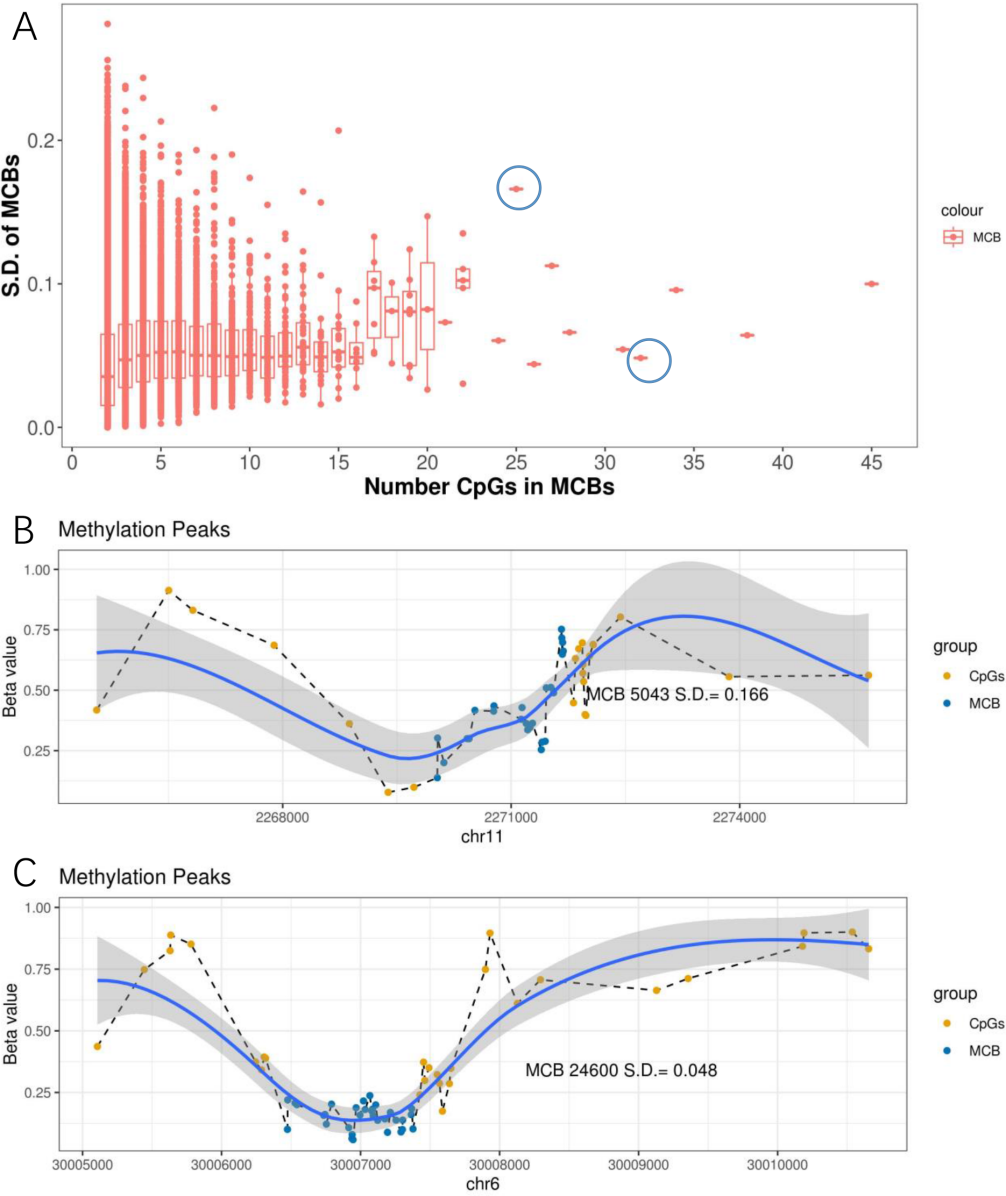
Methylation peaks of CpG sites. (A) Correlation between standard deviation and quantity of CpGs in MCB. The left blue circle indicates the MCB with maximum standard deviation in the top 10 most CpG enriched MCBs, while the right one indicates the low standard deviation. Methylation status in methylated correlated blocks regions, which indicated by two circles, were shown in (B) and (C), respectively. Compared to (C), the MCB located in the uphill of methylation peaks (B) shows larger deviation. The right *Y*-axis indicates the *β* values. Blue dots represent CpGs in MCBs and the yellow ones represent boundaries. Methylation curves were smoothed by local polynomial regression fitting.

We further observed that when the number of CpGs within the MCBs increased, the standard deviation decreased (Fig. 4A). Notably, some MCBs which had high numbers of CpGs also showed high standard deviations. Therefore, we drew the mean *β* value distribution of the CpG sites in the MCBs that had the most enriched CpGs. The methylation curves for the CpG sites showed methylation peaks in the chromosomes. Moreover, MCBs located in the uphill or downhill of the peaks had relatively high standard deviations, while MCBs in the edges of the peaks had low standard deviations. Figures 4B and 4C show the correlation between mean distribution of *β* values in chromosomes and the high and low standard deviations in the top 10 CpG-enriched MCBs in the euchromosome.

### Prognostic capacity of individual CpG sites and methylation correlation blocks

**Figure 5:**
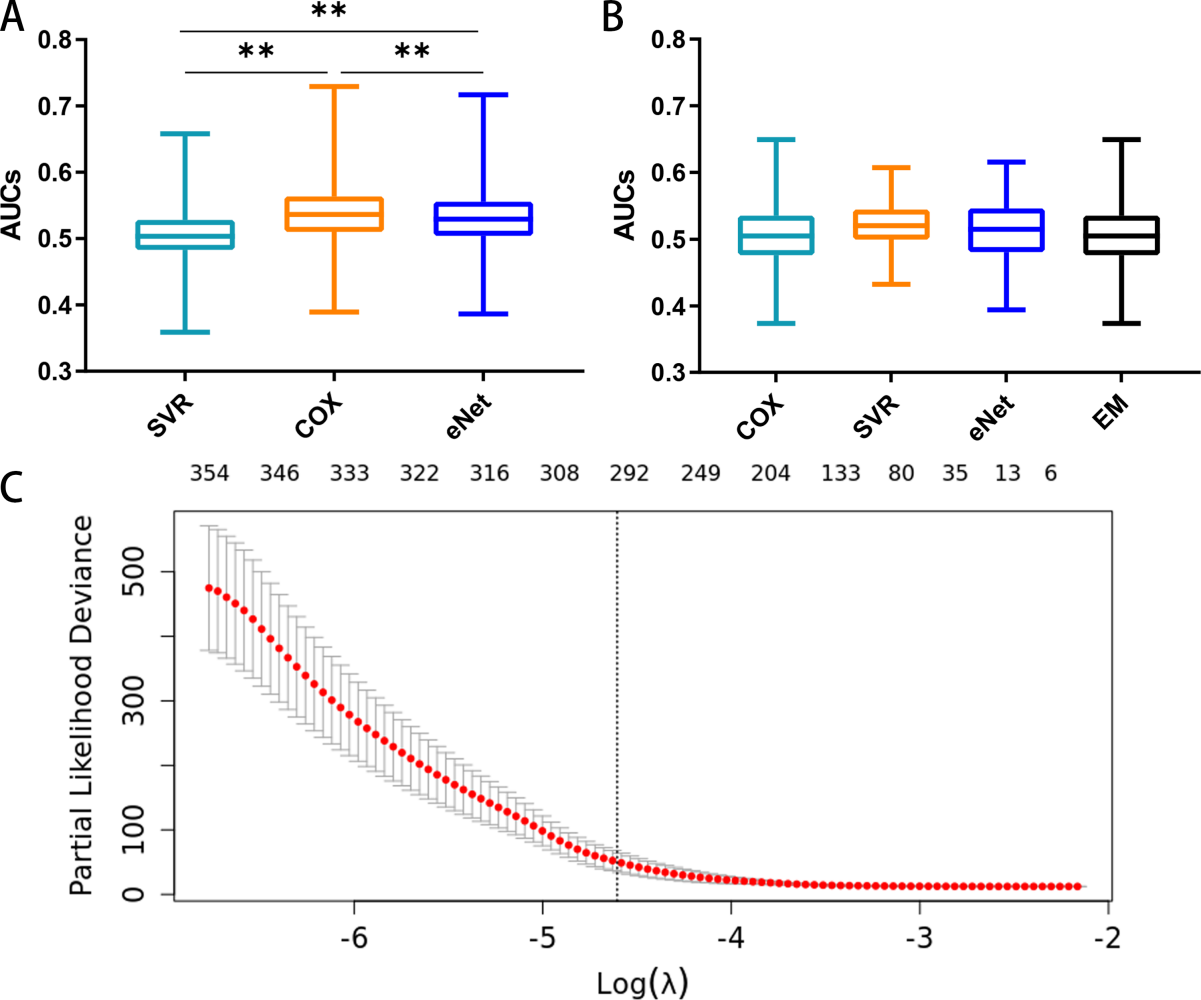
Feature selection and the distribution of prognostic capacity. (A) The distribution of prognostic capacity indicated by AUCs using Cox regression, support vector regression, and elastic-net regression model. The asterisk indicates a significant difference (*p* < 0.01) using the paired *t*-test and analysis of variance. (B) The distribution of prognostic capacity indicated by AUCs using Cox regression, support vector regression, elastic-net regression, and ensemble model after feature selection in the training set. The elastic-net regression and ensemble models were marked as “eNet” and “EM”, respectively. AUCs were calculated by 10 fold cross validation. (C) Feature selection curve based on cox regression using L1 regularization. The curve is shown as a red dotted line, and bars represent the upper and lower standard deviation. The plot shows the curves along the *λ* sequence. Selected *λ* as the threshold is indicated by the vertical dotted lines, MCB preserved indicated as upper numbers.

The prognostic capacities (AUC) of the three MCB-based models, namely elastic-net regression (AUC 0.386–0.717), SVR (AUC 0.359- 0.658), and Cox regression modeling (AUC 0.389–0.729), are shown in Fig. 5A. Cox regression modeling showed elevated performances (*p* < 0.01, paired *t*-test) in the training set (Fig. 5A) when compared to the elastic-net regression and SVR.

We next performed embedded feature selection (L1 Regularization) based on Cox regression with the MCB arithmetic mean. Using a threshold of *λ* = 0.01, we preserved a total of 297 MCBs (Fig. 5C).

After feature selection, we calculated the signature score for individual MCBs in separate models (Cox, SVR, and elastic-net, along with an ensemble model) using 10-fold cross validation. We assayed the AUC and C-index for each MCB in the training set (Table S2). Our results showed that the Cox, SVR, and elastic-net algorithms performed similarly (*p* > 0.05, paired *t*-test) when compared to the ensemble model. The median AUCs in the training set were 0.505, 0.520, 0.515, and 0.505 for the Cox, SVR, elastic-net regression, and ensemble models, respectively. The maximum AUCs in the training set were 0.649, 0.607, 0.616, and 0.649, for the Cox, SVR, elastic-net regression, and ensemble models, respectively. The box plots for the MCB AUCs are shown in Fig. 5B.

### Prognostic capacity of stacking ensemble model

After using the TCGA dataset for MCB identification and model evaluation, we further tested the models in the validation set to assess the effects of heterogeneity between data sets on our models. We ranked the MCBs by the RP method (based on the AUC results of multiple models) to assess their performance in the training and validation sets. After training and validation, the models were selected and finalized. The holdout cohort (103 samples) was used as testing set and only for final validation. We identified the MCBs with the top five RP values in the training and validation sets (Table 1). The MCB-29016-based classifier has the best performance. This MCB-29016-based ensemble classifier correlated with *PSD3* gene (contained 5 CpGs) had AUCs of 0.622 in the training set, 0.773 in the validation set, and 0.698 in the testing set (Table S3).

We also carried out another procedure that combined the TCGA and the GEO datasets. We randomly divided those mixed samples into the training and the testing sets (8:2). The MCBs were identified and selected by L1 penalty using the training set. We further analyzed the performances (AUCs) of multiple models using 10-fold cross validation in the training set. We found that the results using cross validation (training/testing) procedure were similar to that of the training/validation/testing method. The MCB-29016-based classifier was also top ranked (only based on the training set) and show good results in the testing set. The AUC in the training set (AUC 0.658) was slightly higher than that of in the testing set (AUC 0.650). The results are shown in Table S4.

**Figure 6:**
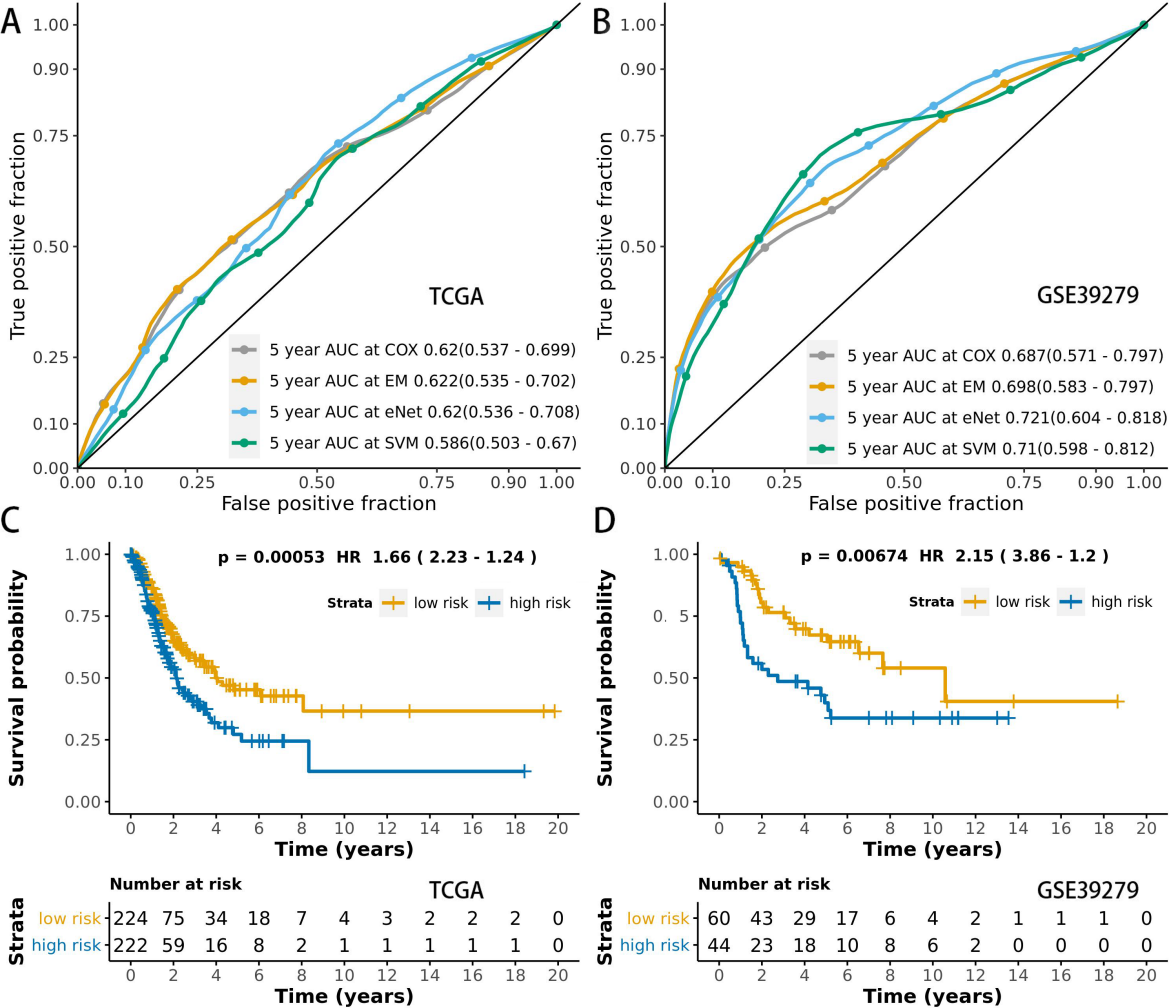
ROCs and survival curves of the models. Time-dependent ROC presents the high sensitivity and specificity for predicting patient’s survival in the training set (A) and testing set (B). We used AUCs (95% confidence interval) at 5 years to assess the prognostic accuracy of Cox regression. The elastic-net regression model and ensemble model are marked as “eNet” and “EM”, respectively. The curves of the ensemble model were similar to that of the Cox regression model. DFS curves of LUAD patients with a low or high risk of death in the training set (C) and testing set (D) are plotted according to a prognosis score calculated from a panel of CpGs in MCB. The Kaplan–Meier curves of DFS in LUAD patients with a low or high risk of DE are shown. The *p* values were calculated using the log-rank test. AUC values were rounded to three decimal places.

We used time-dependent ROC analysis with 5-year follow-up times for the training and testing sets to assess the prognostic accuracy of the MCB-29016-based classifier (Figs. 6A and 6B). The ensemble model had an AUC of 0.622 in the training set (LUAD in TCGA) and an AUC of 0.698 in the testing set. The distribution of ROC curves did not significantly vary across the different prediction models (*p* > 0.05, bootstrap percentile method).

**Table 1:** Results of RP for individual MCBs.

**MCB No.**	**Location**	**Length**	**CpGs**	**Genes**	**RP**
29016	chr8: 18541446–chr8: 18541627	181	5	*PSD3*	14.245
12936	chr17: 38465281–chr17: 38465510	229	7	*RARA*	26.414
24635	chr6: 30094980–chr6: 30095802	822	27	*Not Available*	26.867
952	chr1: 27683139–chr1: 27683501	362	5	*MAP3K6*	26.890
17010	chr2: 73429308–chr2: 73430374	1066	10	*NOTO*	27.231

We used multivariate Cox regression analysis of the training set to further reveal the associations between the MCB-based classifier, clinicopathology, and DFS (Table 2). We additionally found that the MCB-29016-based classifier performed well in differentiating low-risk and high-risk groups in Kaplan–Meier analyses of patient DFS and in associated log-rank tests with significant *p* values in the training sets (Fig. 6C), and in demonstrating the significant prognostic utility of MCB signatures in LUAD. To confirm that the MCB-based classifier had similar prognostic value across different populations, we applied it to the validation set. And then tested the classifiers in the testing set of 103 patients from the GEO database (GSE39279, Table S6). We noted similar results in the testing set (*p* < 0.01, Fig. 6D).

**Table 2:** Association of clinicopathological characteristics with disease-free survival in the training set.

Label	Hazard ratio (95% CI)	Multivariate *p* value
Smoking history	1.02 (0.668–1.56)	0.72
Pathologic stage	1.44 (1.25–1.67)	0.007^**^
TNM stage: T	1.39 (1.17–1.64)	0.061
TNM stage: N	1.17 (1.02–1.35)	0.693
TNM stage: M	1.01 (0.933–1.09)	0.598
Risk score	2.28 (1.19–4.37)	0.021^*^

**Notes.**

Significant codes: <0.01**, <0.05*.

**Figure 7:**
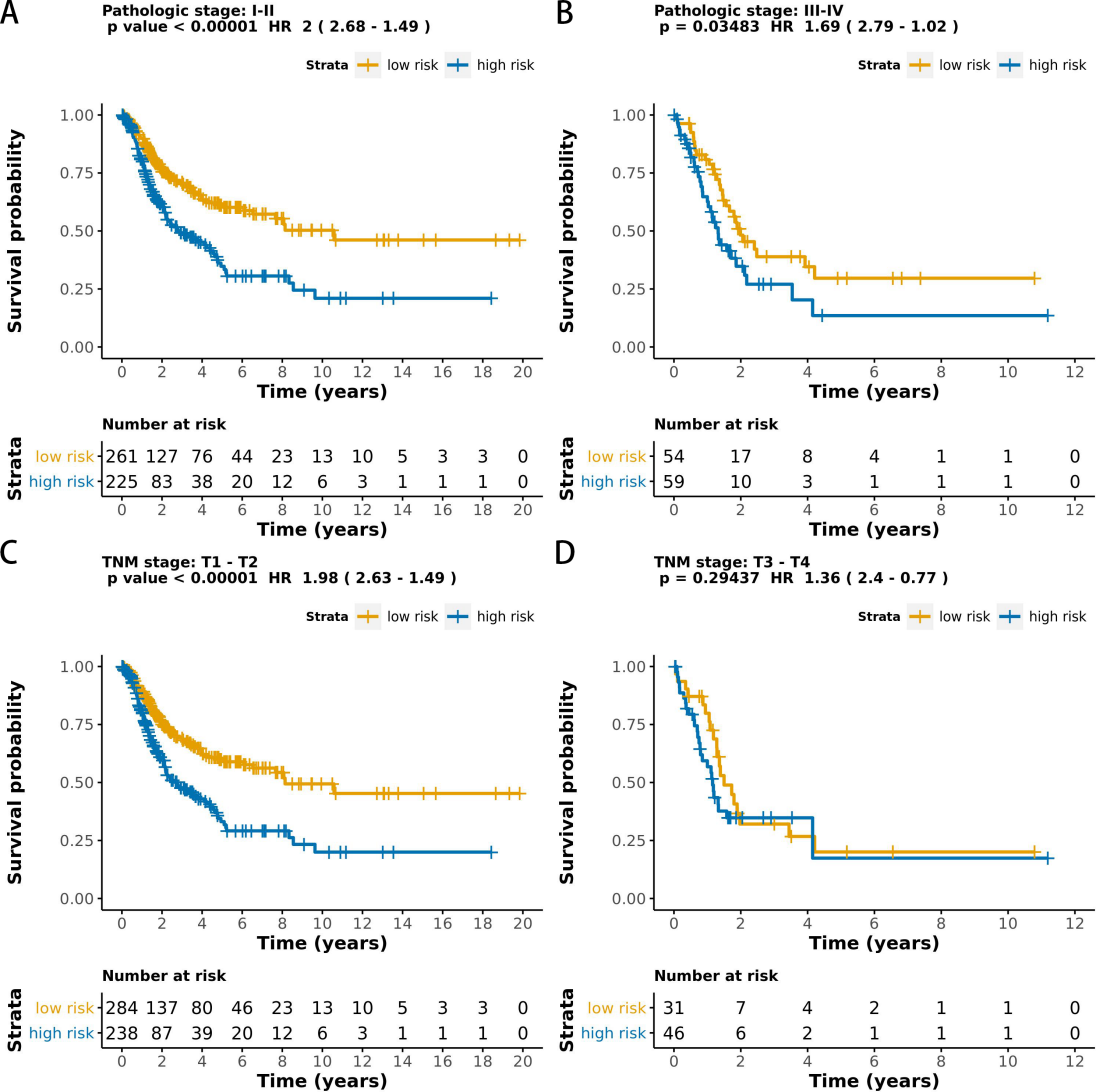
Kaplan-Meier survival analysis for all 599 patients with LUAD according to the MCB-29016-based classifier stratified by pathologic stage and tumor size. The *p* values were calculated using the log-rank test.

**Figure 8:**
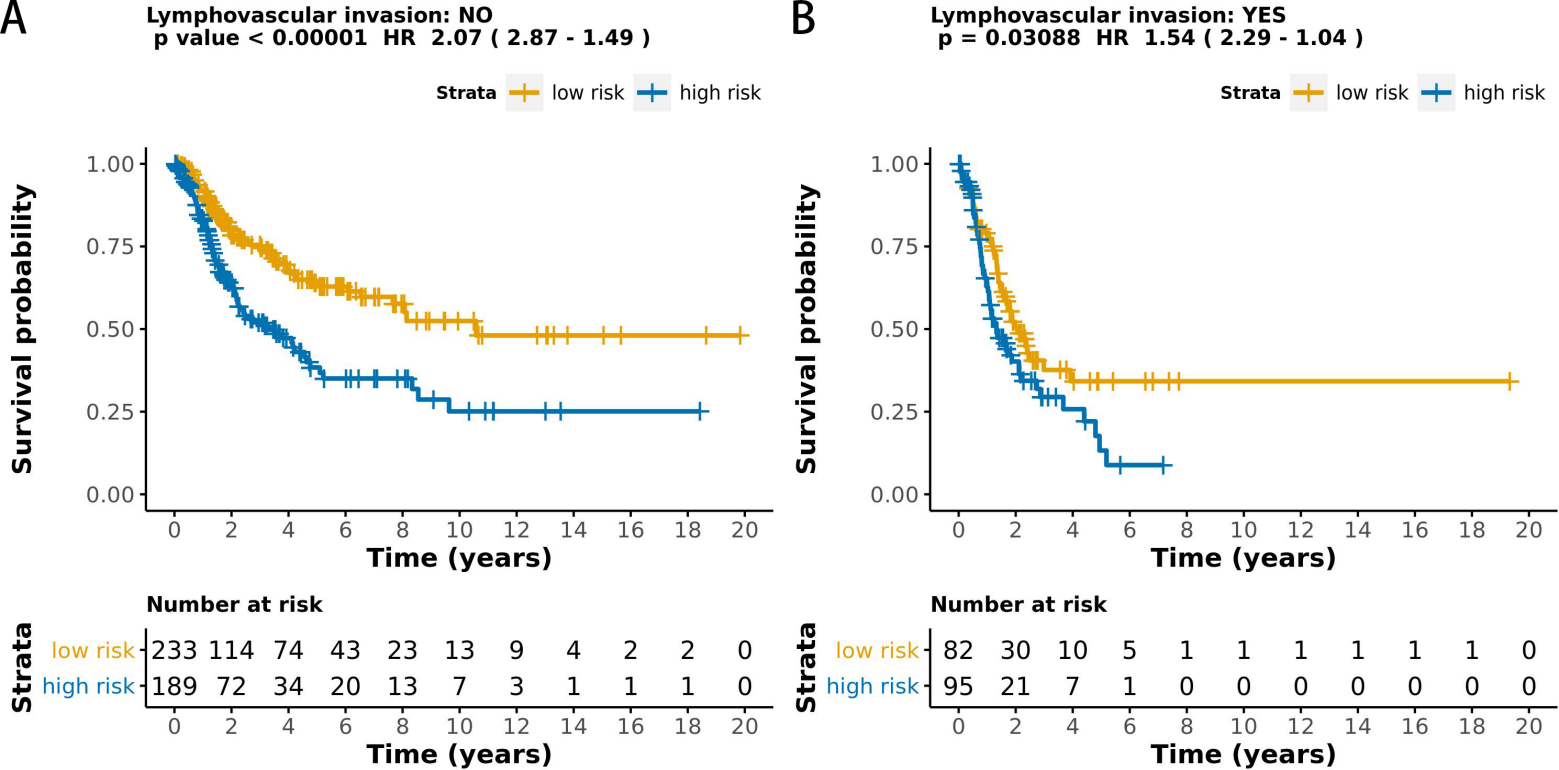
Kaplan-Meier survival analysis for all 599 patients with LUAD according to the MCB-29016-based classifier stratified by lymphovascular invasion. The *p* values were calculated using the log-rank test.

The MCB-29016-based classifier showed significantly high prognostic accuracy across the entire dataset and showed the potential to be an independent prognostic factor along with clinicopathology. When stratified by clinicopathological risk factors, the MCB-29016-based ensemble classifier functioned as a clinically and statistically significant prognostic model. The prognostic value of the MCB-29016-based classifier presented very positive results in early stage LUAD. The results showed a statistical significance of *p* < 0.001 (log-rank test) in pathological stages 1–2, tumor-node-metastasis (TNM) stages T1-T2 (Fig. 7), and N0 (Fig. 8). Therefore, we concluded that the MCB-29016-based classifier can add prognostic value to clinicopathological prognostic features.

To provide easy access when using this stacking ensemble system, we further established a prognostic nomogram along with the online prediction tool using the shiny R framework based on the MCB-29016-based classifier and the clinicopathological data of all LUAD patients (Fig. S3). The online resources for the nomogram and prediction application (Zhang et al., 2017; Zhang & Kattan, 2017) can be found at https://enmcb.bioinfo.xin.

## Discussion

In this study, we developed and validated a novel prognostic tool based on MCBs that improved DFS prediction in LUAD patients. After screening high-throughput CpG and MCB data, our proposed classifiers can predict the DFS of LUAD patients in the training set and also in the testing set. Furthermore, our results showed that this tool can successfully categorize patients into high-risk and low-risk DFS groups.

Our analysis of CpG co-methylation in cancers was based on the theoretical framework of linkage disequilibrium (Ardlie, Kruglyak & Seielstad, 2002), which was developed to model the co-segregation of adjacent genetic variants in human chromosomes. There have been a number of studies related to methylation haplotypes (Itabashi et al., 2018; Seoighe, Tosh & Greally, 2018), epi-alleles (Brocklehurst et al., 2018; Hellesoy & Lorens, 2015), and epi-haplotypes (Xu et al., 2018), but they mostly focused on specific genomic regions or cell/tissue types. Due to the locally coordinated activities of methyltransferase enzymes, including DNA methyltransferase 1, DNA methyltransferase 3 A/B, and ten to eleven translocation proteins, adjacent CpG sites on the same DNA molecules can share similar methylation statuses. However, discordant CpG methylation has been observed, especially in cancer (Guo et al., 2017). In our results, these short and punctuated MCBs were widespread in the human genome, indicating that there were discrete entities of epigenetic regulation in tumors. This phenomenon can be further harnessed to improve the robustness and sensitivity of DNA methylation analysis; for example, incorporating the deconvolution of data from heterogeneous samples (Guo et al., 2017).

We also found that MCB lengths were relatively short and independent of genome size. The linkage disequilibrium level typically decayed across the range of hundreds of kilobases to megabases (Guo et al., 2017; Seoighe, Tosh & Greally, 2018). In contrast, CpG co-methylation depended on DNA methyltransferases and demethylases, which tended to have much lower processivity, and, in the case of hemi-methyltransferases, much lower fidelity, when compared with DNA polymerases (Guo et al., 2017). Therefore, the CpG methylation levels decayed across a much shorter distance of tens to hundreds of bases. Our results showed that the mean MCB length was 204 bp, which supported this concept.

Our pipeline allowed users to integrate multiple CpG blocks into one panel, which has prognostic potential. In our study, we identified MCBs across the full genome and then proposed a block-level metric to systematically discover informative markers. Applying such an analytic framework can produce accurate predictions of cancer status in clinical plasma samples from cancer patients, as mammalian CpG methylation is a relatively stable epigenetic modification. Several previous attempts used machine learning tools for data mining to predict survival. These attempts included the use of Cox (Ma et al., 2020), SVR (Das, 2010), elastic-net (Guo et al., 2017), deep learning (Katzman et al., 2018), and ensemble models (Bonato et al., 2011; Hothorn et al., 2006; Pourhoseingholi, Kheirian & Zali, 2017). These different attempts identified strong correlations. A previous study used LASSO modelling to calculate the 5-year survival rate with an AUC of 0.75 (Ma et al., 2020). Another study used cutaneous melanoma data and a linear model to predict the survival rate with an AUC of 0.822 (Guo et al., 2019). Linear models are faster and may be more suitable for methylating 450k microarrays since probes mainly show three distinct methylation states (Triche Jr et al., 2013). However, due to insufficient research on DFS and methylated regions, current survival models have been largely based on several methylated sites instead of regions (Capper et al., 2018; Guo et al., 2019; Ma et al., 2020). To overcome this, studies have found that co-methylation CpGs or methylation clusters can reflect differences in environmental conditions (Liu et al., 2014b). These findings can be used to develop diagnostic and prognostic prediction methods by summing up all the neighboring CpGs in one local methylation profile (Guo et al., 2017; Liu et al., 2014b; Xu et al., 2017). However, methylation peaks in MCBs can shift both vertically and horizontally (Konno et al., 2019). These shifts and varieties also need to be considered for intro CpGs and detected by models that are more complex than sum or mean methylation values. Among the models used in our pipeline, the classic Cox model was easy to interpret, elastic-net methods could be used for feature selection, and SVR models used for all CpGs gave elevated performance. Moreover, using an ensemble is a proven strategy for integrating multiple models with different prospects and improving the accuracy of the models (Bonato et al., 2011; Pourhoseingholi, Kheirian & Zali, 2017). An ensemble model can be used to expand the use of methylation data, although it was unable to further improve the performance of single models in our study. Our pipeline in R and Bioconductor was faster and easier to implement with parallel computation. To the best of our knowledge, this is the only available ensemble machine learning pipeline for survival analysis that is based on methylated site regions. Our study’s objective was to compare prediction methods to standard statistical models in order to predict DFS using methylated region data.

Our study found that MCB-based models had elevated performances. Previous studies have identified multiple markers that are differentially regulated in LUAD with improved accuracy. This research has shown that the evaluation of a DNA methylation panel is a highly sensitive method for detecting cancer (Diaz-Lagares et al., 2016; Kim & Kim, 2015; Saito & Suyama, 2015; Shimizu et al., 2013). Lehmann-Werman et al. (2016) also demonstrated a superior sensitivity to multiple adjacent CpGs when detecting tissue-specific signatures based on the genome’s methylation status. These findings and our results support the use of multiple adjacent CpGs’s methylation status as a novel way to increase specificity because the methylation level of individual CpG sites may be partially limited by technical noise and sensitivity when measuring single CpG methylation. Moreover, previous studies have identified markers that were shown to be associated with prognoses or therapeutic outcomes in cancer patients, with AUCs ranging from 0.6–0.8 regarded as acceptable performances (Zhang et al., 2013). The use of the ensemble model allowed us to take advantage of multiple models, and it was relatively more stable than using a single CpG or model.

We also explored the biological function of the MCBs used in our classifier. The methylation of CpG islands within the promoters (Sun, Liu & Xu, 2018) and/or the coding region (Arechederra et al., 2018) of the tumor suppressor genes was one of the most frequently acquired epigenetic changes during lung cancer pathogenesis and is usually associated with transcriptional gene down-regulation (Kim & Kim, 2015). In particular, the *PSD3* gene has been shown to be associated with cancer prognosis or therapeutic outcomes. A gene related to *TP53* (Xie et al., 2012), one of the best cancer markers, has been detected and is associated with many types of cancer (Bhatlekar, Fields & Boman, 2014) including ovarian cancer (Yuan et al., 2015), breast cancer (Yang et al., 2017), and lung cancer (Liu et al., 2014a).

Our MCB-based classifier provides diagnostic value that complements pathological risk factors and can be used to define subgroups with different risks in LUAD patients. However, the prognostic value of MCB may need to be further refined using more advanced models. The panel reported here has limited generalization ability since censored survival data is not precise when compared to binary data, and the distribution of MCBs in patients depended on the mutation rate, frequency of meiotic recombination, an effective population size, and demographic history. These MCBs may be different in larger cohorts and may be susceptible to the inherent biases of such a study framework. Clearly, our results should be further validated by future prospective studies in multi-center clinical trials.

## Conclusion

In conclusion, we have shown that an MCB-based diagnostic tool using the ensemble strategy effectively classifies LUAD patients, especially in the early stages, into low and high risk groups and predicts the survival of those patients. Our model complements the advanced use of DNA methylation profiles for survival analysis and supports the potential utilization of MCBs as signatures.

##  Supplemental Information

10.7717/peerj.10884/supp-1 Figure S1 The distribution of the length of MCBs in individual MCBs

10.7717/peerj.10884/supp-2 Figure S2 The distribution of the quantity of CpGs in individual MCBs

10.7717/peerj.10884/supp-3 Figure S3 Screenshot of our prediction tool and the prognostic nomogram based on MCB-29016-based classifier To use this tool, one may input the *β* value of CpGs by using sliders at the left then the risk score is calculated instantly. The nomogram has a reference line for reading corresponding scoring points. Once the reader sums all the scoring points, the predicted values (survival risks) can be read at the bottom lines. For more details (nomogram usage) please read (Zhang et al., 2017; Zhang & Kattan, 2017).

10.7717/peerj.10884/supp-4 Table S1 Clinical Characteristics of the training and validation sets

10.7717/peerj.10884/supp-5 Table S2 The association between MCB-based classifiers and DFS in the training set The elastic-net regression model is marked as “eNet”.

10.7717/peerj.10884/supp-6 Table S3 The association between MCB-based classifiers and DFS in the training, validation set and testing set The ranking was based on the AUC results of MCB-based classifiers in the training, validation and testing sets.

10.7717/peerj.10884/supp-7 Table S4 The association between MCB-based classifiers and DFS in the training and testing set using another procedure We also carried out another procedure that combined the TCGA and the GEO datasets. We randomly divided those mixed samples into the training and the testing sets (8:2). The MCBs were identified and selected by the L1 penalty using the training set. We further analyzed the performances (AUCs) of multiple models using 10-fold cross validation in the training set. We found that the results using cross validation (training/testing) procedure were similar to that of the training/validation/testing method. MCB-29016-based classifiers were also top ranked (only based on the training set) and show good results in the testing set. The AUC in the training set (AUC 0.658) was slightly higher than that of in the testing set (AUC 0.650). The ranking was based on the AUC results of MCB-based classifiers in the training and testing sets.

10.7717/peerj.10884/supp-8 Table S5 Packages used to perform the experiments and the configuration of the parameters and execution environment

10.7717/peerj.10884/supp-9 Table S6 The univariate and multivariate Cox regression analysis of the validation and testing sets (GEO database)

## Additional Information and Declarations

### Data Availability

The following information was supplied regarding data availability: Data are available at TCGA (LUAD) and NCBI GEO (GSE39279). EnMCB (version 1.2.2) package in R and the source code are freely available at GitHub: https://github.com/whirlsyu/EnMCB/. Analysis scripts are also freely available at GitHub: https://github.com/whirlsyu/EnMCB_analysis_for_lung_adenocarcinoma.
