# Supplementary figures and images for: Predicting lung adenocarcinoma disease progression using methylation-correlated blocks and ensemble machine learning classifiers
Source: PeerJ. 2021 Feb 16;9:e10884. doi: 10.7717/peerj.10884 (PMC7894106; doi:10.7717/peerj.10884)

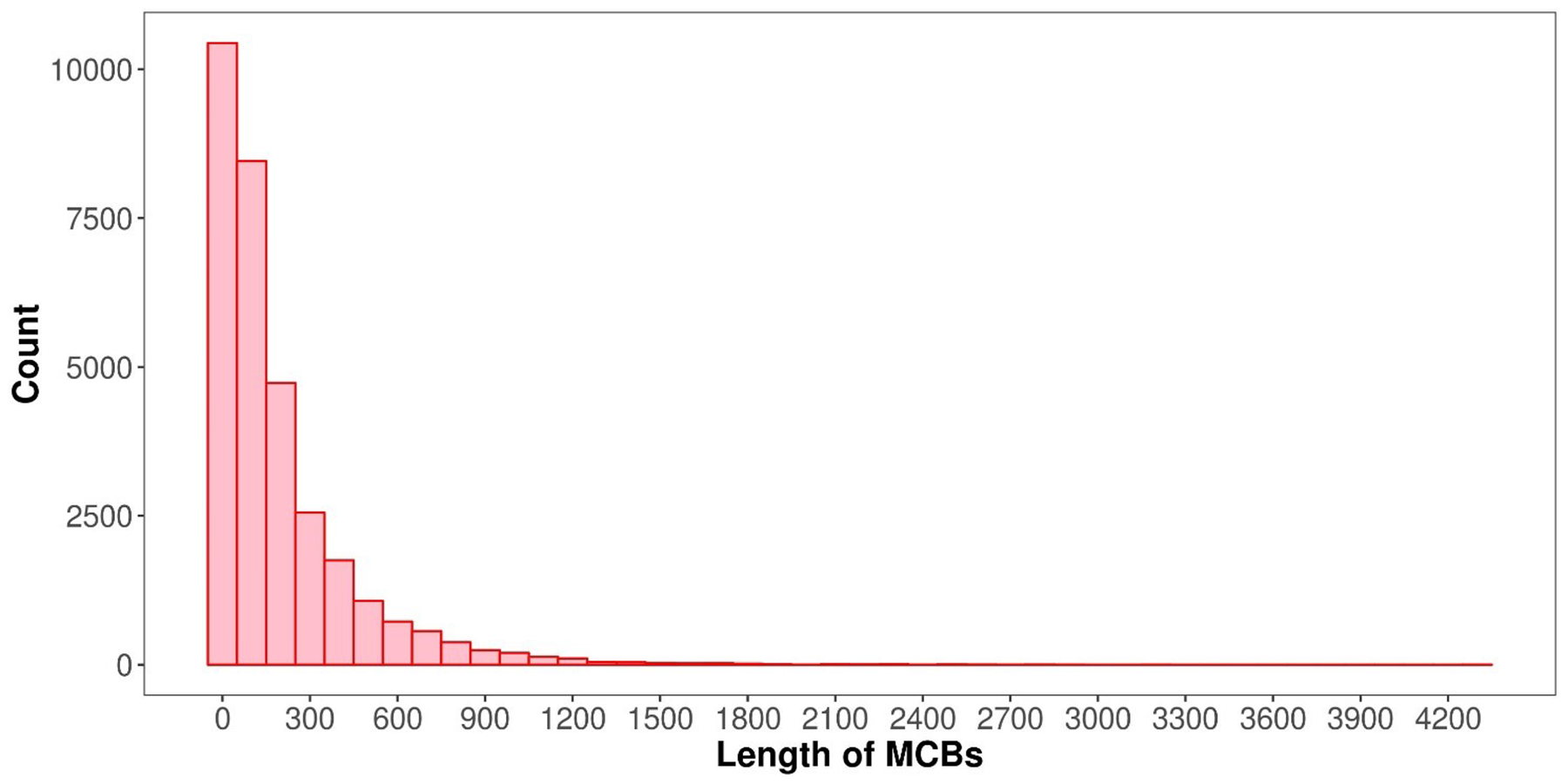

Supplement: Figure S1 [file peerj-09-10884-s001.png]

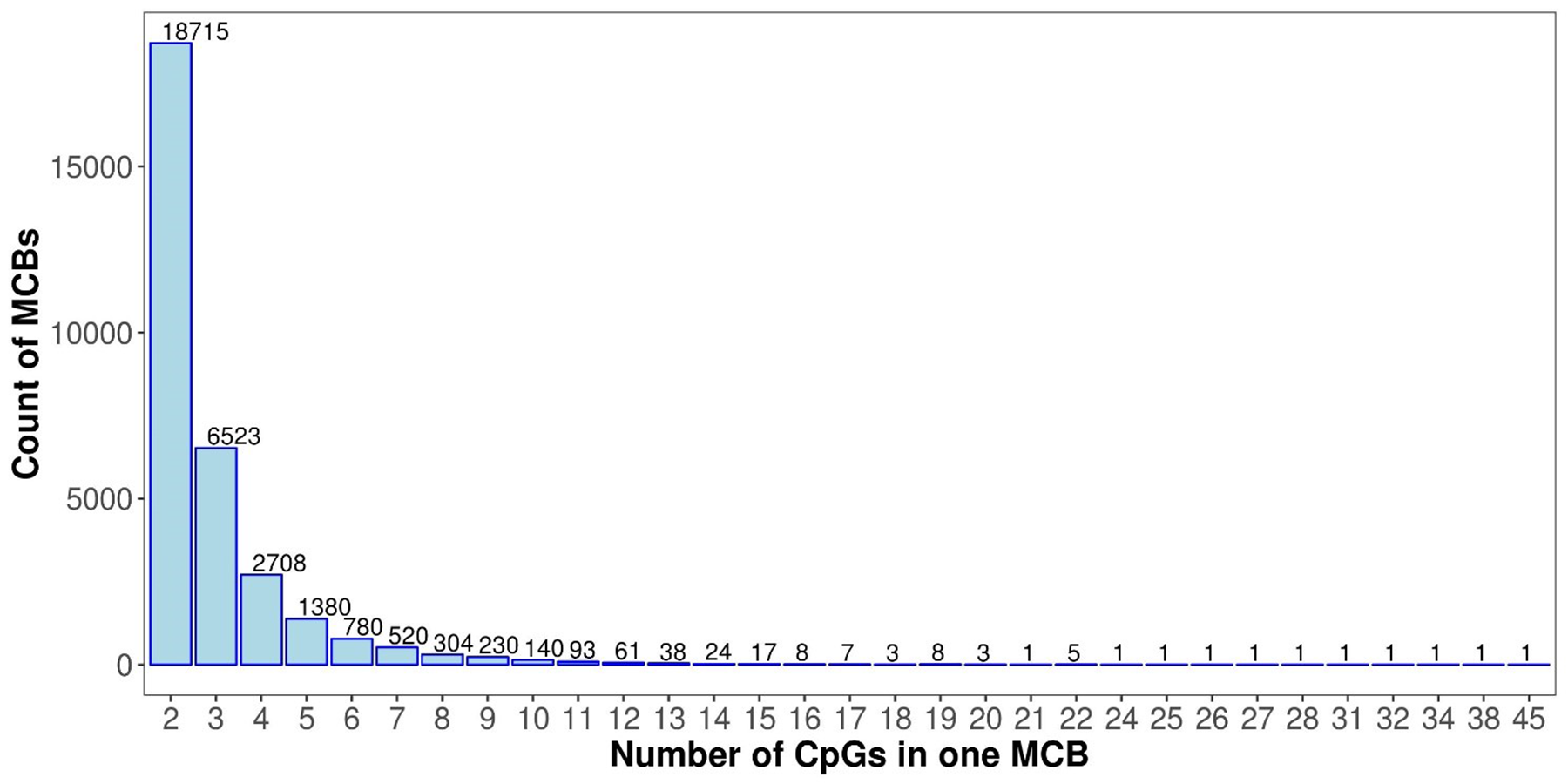

Supplement: Figure S2 [file peerj-09-10884-s002.png]

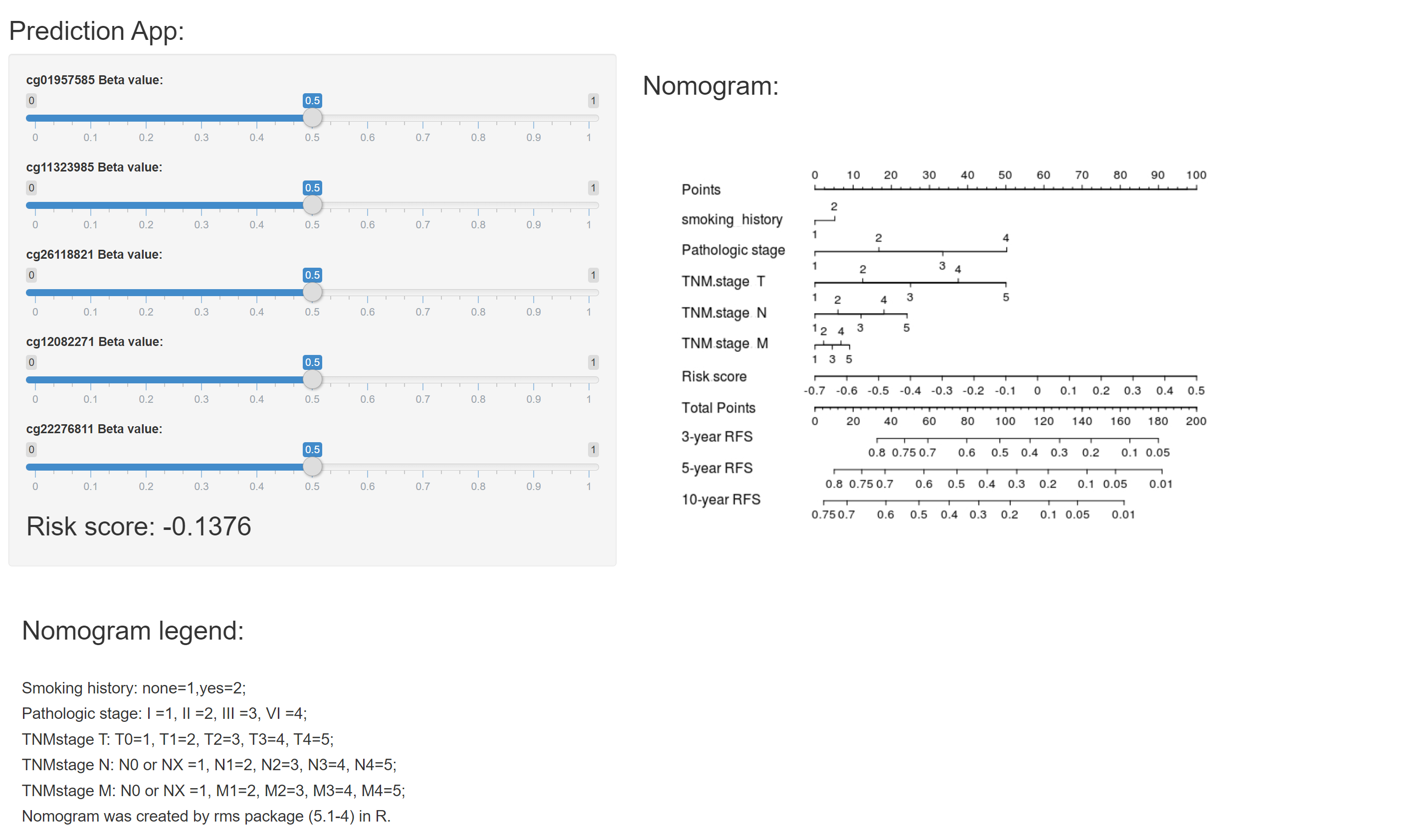

Supplement: Figure S3 — To use this tool, one may input the β value of CpGs by using sliders at the left then the risk score is calculated instantly. The nomogram has a reference line for reading corresponding scoring points. Once the reader sums all the scoring points, the predicted values (survival risks) can be read at the bottom lines. For more details (nomogram usage) please read (Zhang et al., 2017; Zhang & Kattan, 2017). [file peerj-09-10884-s003.png]
