# Supplementary material for: Predicting lung adenocarcinoma disease progression using methylation-correlated blocks and ensemble machine learning classifiers
Source: PeerJ. 2021 Feb 16;9:e10884. doi: 10.7717/peerj.10884 (PMC7894106; doi:10.7717/peerj.10884)
Supplement: Table S1 [file peerj-09-10884-s004.docx]

**Supplementary Table 1.** Clinical Characteristics of the TCGA and GEO data sets

| Characteristic | Training Cohort  (TCGA, n= 461) ^*^ |  | Subset From Training Cohort for DFS Analysis (TCGA, n= 444) |  | Validation and testing Cohort  (GEO, n= 155) |  |
| --- | --- | --- | --- | --- | --- | --- |
|  | No. % |  | No. % |  | No. % |  |
| Age, years |  |  |  |  |  |  |
| Median | 65.5 |  | 66 |  | 65 |  |
| Mean | 65.12 |  | 65.08 |  | 65.11 |  |
| Range | 33-88 |  | 33-88 |  | 40-85 |  |
| Sex |  |  |  |  |  |  |
| Male | 215 47 |  | 207 47 |  | 76 49 |  |
| Female | 246 53 |  | 237 53 |  | 79 51 |  |
| Smoking history |  |  |  |  |  |  |
| Current or former smoker | 380 82 |  | 366 82 |  | 129 83 |  |
| Nonsmoker | 67 15 |  | 64 15 |  | 23 15 |  |
| Unknown | 14 3 |  | 14 3 |  | 3 2 |  |
| Disease stage |  |  |  |  |  |  |
| I | 250 54 |  | 241 54 |  | 118 76 |  |
| II | 113 25 |  | 109 25 |  | 18 12 |  |
| III | 73 16 |  | 70 16 |  | 16 10 |  |
| IV | 20 4 |  | 20 4 |  | 3 2 |  |
| Unknown | 5 1 |  | 4 1 |  | 0 0 |  |
| TNM stage: T |  |  |  |  |  |  |
| T1 | 157 34 |  | 153 34 |  | 80 52 |  |
| T2 | 244 53 |  | 235 53 |  | 54 34 |  |
| T3 | 41 9 |  | 37 8 |  | 10 7 |  |
| T4 | 16 4 |  | 16 4 |  | 11 7 |  |
| Tx | 3 0 |  | 3 1 |  | 0 0 |  |
| TNM stage: N |  |  |  |  |  |  |
| N0 | 301 66 |  | 290 65 |  | 132 85 |  |
| N1 | 84 18 |  | 82 18 |  | 13 8 |  |
| N2 | 65 14 |  | 62 14 |  | 10 7 |  |
| N3 | 1 0 |  | 1 0 |  | 0 0 |  |
| Nx | 9 2 |  | 9 2 |  | 0 0 |  |
| Unknown | 1 0 |  | 0 0 |  | 0 0 |  |
| TNM stage: M |  |  |  |  |  |  |
| M0 | 298 65 |  | 283 64 |  | 152 98 |  |
| M1 | 19 4 |  | 19 4 |  | 3 2 |  |
| Mx | 140 30 |  | 142 32 |  | 0 0 |  |
| Unknown | 4 1 |  | 0 0 |  | 0 0 |  |
| Recurrence |  |  |  |  |  |  |
| Yes | 122 26 |  | 182 41 |  | 68 44 |  |
| NO | 339 74 |  | 262 59 |  | 87 55 |  |
| Unknown | 0 0 |  | 0 0 |  | 0 0 |  |
| DFS, years |  |  |  |  |  |  |
| Median | 1.41 |  | 1.43 |  | 3.68 |  |
| Mean | 2.05 |  | 2.05 |  | 4.56 |  |
| Range | 0-19.84 |  | 0.01-19.84 |  | 0.05-18.64 |  |

* 492 methylation profiles from total 461 patients. Profiles include tumor and adjacent normal tissues.
